# Supplementary material for: Combining genomics and epidemiology to investigate a zoonotic outbreak of rabies in Romblon Province, Philippines
Source: Nat Commun. 2024 Dec 30;15:10753. doi: 10.1038/s41467-024-54255-5 (PMC11685615; doi:10.1038/s41467-024-54255-5)
Supplement: Supplementary file 1 — Supplementary Information [file 41467_2024_54255_MOESM1_ESM.pdf]

## Supplementary Files:

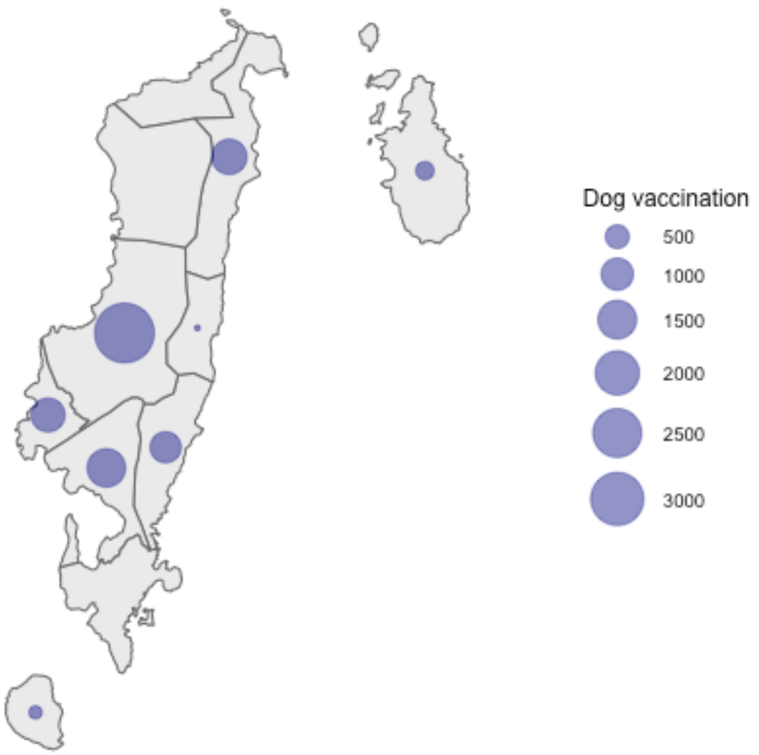


**Supplementary Fig. S1. Number of dogs vaccinated per municipality in Romblon Province (Sept 2022-Sept 2023).**

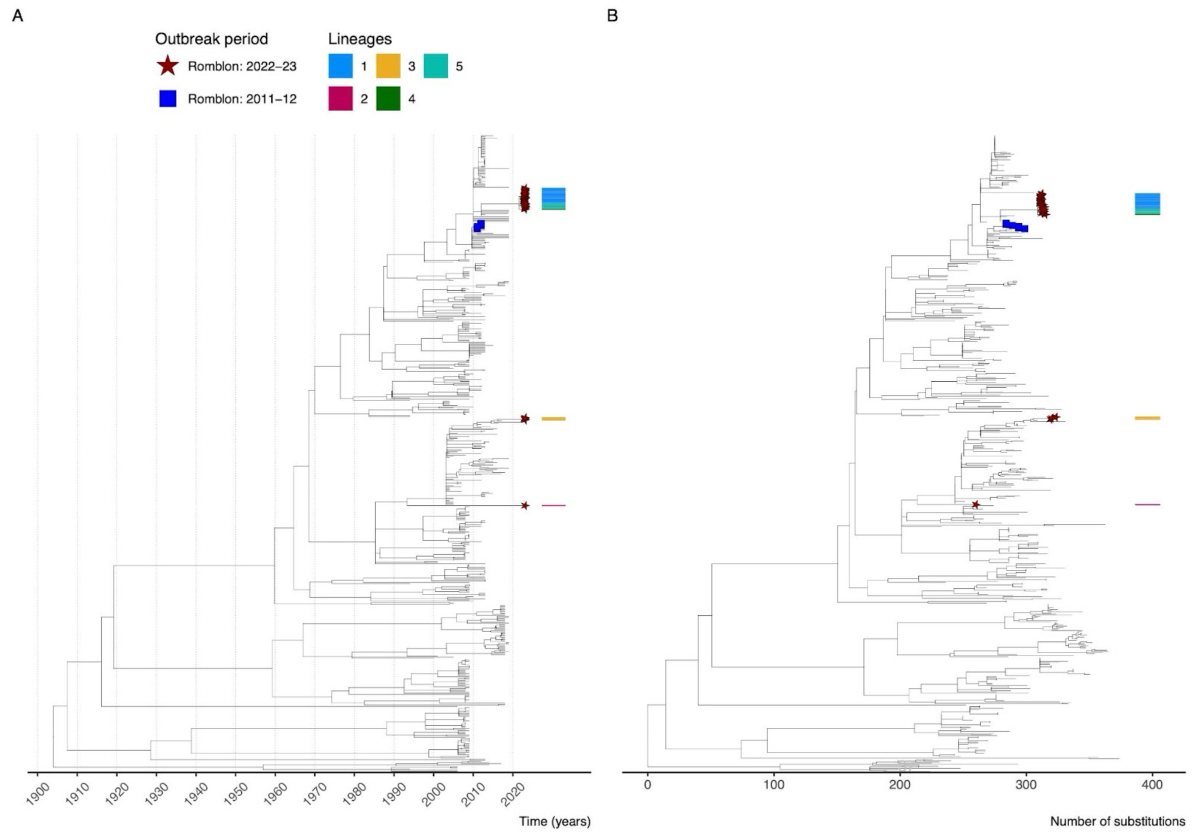


**Supplementary Fig. S2. Time-scaled and substitution-scaled phylogenies from publicly available Philippines RABV sequences.** A) Time-scaled and B) substitution-scaled maximum likelihood trees of 518 sequences (211-11797bp) from the Philippines spanning 1998 to 2023. The phylogenetic placement of Romblon cases from historical (2011-12) and current (2022-23) outbreaks are highlighted, as are the genetic lineages described in the main text. The top cluster from the 2022-23 Romblon outbreak represents the cluster A1 as shown in Figure 3, while the middle cluster is C1 and the lower cluster is the human case B1.

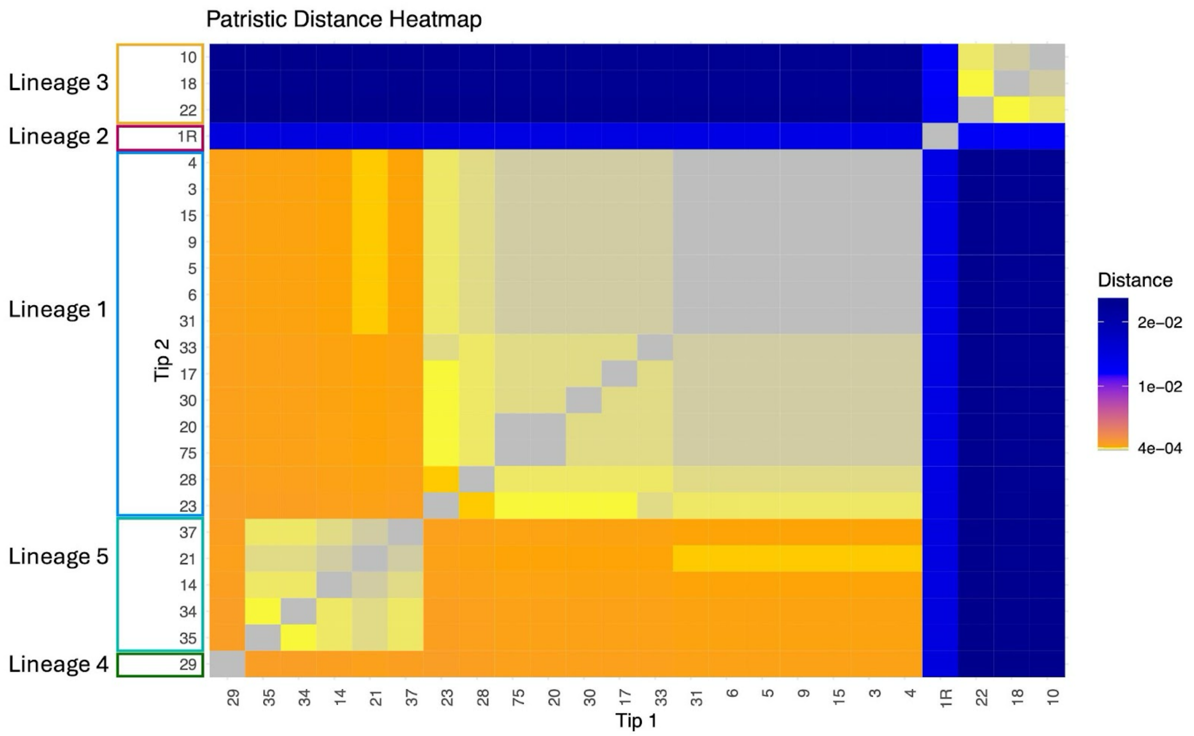


**Supplementary Fig. S3. Patristic distance heatmap of Romblon sequences.** The heatmap illustrates the genetic distances between sequences from Romblon, with distances calculated using the patristic method. The colour gradient runs from grey to yellow to dark blue with shades of blue representing higher genetic distances, indicating less similarity, while yellow/orange shades indicate smaller distances. The transition from yellow to orange marks the 0.0004 threshold used to delineate lineages, which are annotated on the y axis.

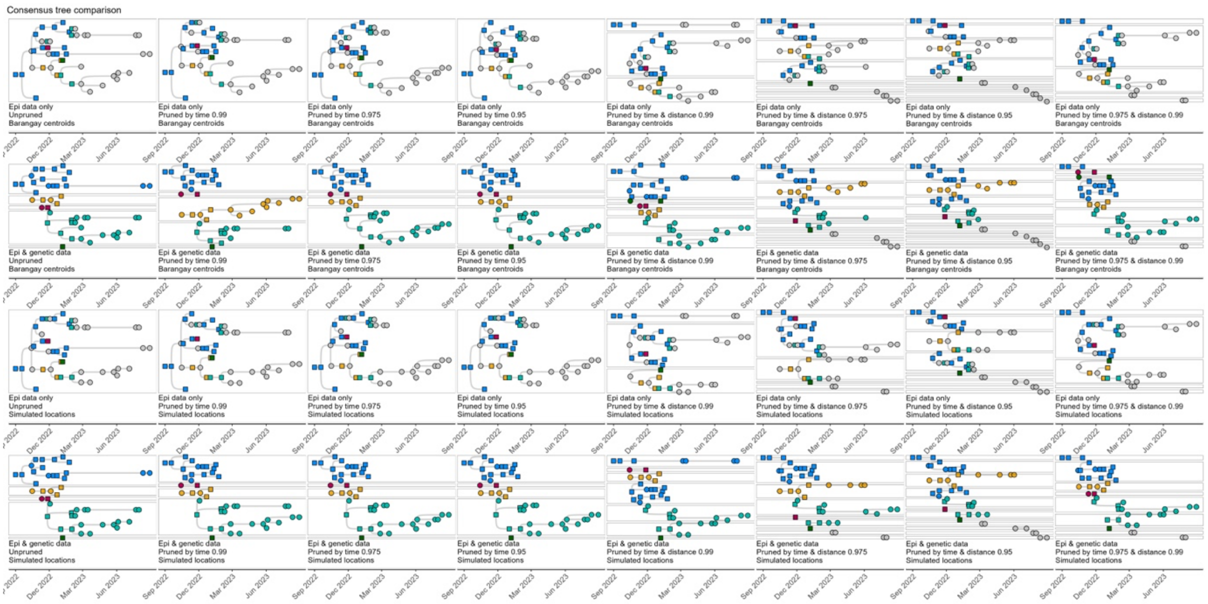


**Supplementary Fig. S4. Consensus transmission tree reconstructions under different pruning thresholds and assumptions about case locations.** Row 1) trees constructed using epidemiological data only and *barangay* centroids to represent case locations; Row 2) trees constructed from epidemiological data consistent with phylogenetic assignments (according to the three introductions

and subsequent divergence of cluster 1 into three genetic lineages, with *barangay* centroid locations); Rows 3) and 4) trees constructed as per Rows 1 and 2 but using case locations simulated in proportion to human population density. Columns 1-8 represent pruning thresholds, specifically 1) unpruned, 2-5) pruned by the 99th, 97.5th and 95th percentile of the serial interval; and 5-7) by the 99th, 97.5th and 95th percentiles of the serial interval and dispersal kernel, and 8) by the 97.5th and 99th percentiles of the serial interval and the dispersal kernel respectively. Squares represent sequences coloured according to lineage, and circles represent detected cases without sequences, coloured by lineage when assigned.

# Supplementary Tables:

**Table S1. Whole genome sequences used in the phylogeography**

| Case ID | Lineage | Cluster | Municipality | Species | Collection Date |
|---|---|---|---|---|---|
| 9 | 1 | 1 | Alcantara | *Canis familiaris* | 01/10/2022 |
| 4 | 1 | 1 | Alcantara | *Canis familiaris* | 21/10/2022 |
| 3 | 1 | 1 | Santa Maria | *Canis familiaris* | 21/11/2022 |
| 5 | 1 | 1 | Odiongan | *Canis familiaris* | 24/11/2022 |
| 6 | 1 | 1 | Alcantara | *Canis familiaris* | 26/11/2022 |
| 15 | 1 | 1 | Odiongan | *Canis familiaris* | 11/12/2022 |
| 75 | 1 | 1 | Santa Maria | *Canis familiaris* | 12/12/2022 |
| 14 | 5 | 1 | Odiongan | *Canis familiaris* | 28/12/2022 |
| 20 | 1 | 1 | Santa Maria | *Canis familiaris* | 20/01/2023 |
| 17 | 1 | 1 | Odiongan | *Canis familiaris* | 13/01/2023 |
| 21 | 5 | 1 | San Agustin | *Canis familiaris* | 20/01/2023 |
| 23 | 1 | 1 | Santa Maria | *Canis familiaris* | 02/02/2023 |
| 29 | 4 | 1 | San Andres | *Canis familiaris* | 07/02/2023 |
| 28 | 1 | 1 | Santa Maria | *Canis familiaris* | 07/02/2023 |
| 33 | 1 | 1 | Santa Maria | *Canis familiaris* | 14/02/2023 |
| 31 | 1 | 1 | Odiongan | *Canis familiaris* | 09/02/2023 |
| 30 | 1 | 1 | Odiongan | *Canis familiaris* | 16/02/2023 |
| 34 | 5 | 1 | Odiongan | *Canis familiaris* | 28/02/2023 |
| 35 | 5 | 1 | Odiongan | *Canis familiaris* | 01/03/2023 |
| 37 | 5 | 1 | San Agustin | *Canis familiaris* | 01/03/2023 |
| 1-R | 2 | 2 | Santa Maria | *Homo sapiens* | 02/02/2023 |
| 10 | 3 | 3 | San Augustin | *Canis familiaris* | 19/12/2022 |
| 18 | 3 | 3 | San Agustin | *Canis familiaris* | 22/01/2023 |
| 22 | 3 | 3 | San Agustin | *Canis familiaris* | 31/01/2023 |

**Table S2. GenBank accession numbers for sequences from rabies samples collected in Tablas Island, Romblon from 2022-2023.**

| Sequin file name | Local reference ID | Accession number |
|---|---|---|
| 4B-23-06.sqn | 4B-23-06 | PP858749 |
| 4B-23-05.sqn | 4B-23-05 | PP858750 |
| Z-22-119.sqn | Z-22-119 | PP858751 |
| 4B-23-02.sqn | 4B-23-02 | PP858752 |
| 4B-23-01.sqn | 4B-23-01 | PP858753 |
| 4B-22-44.sqn | 4B-22-44 | PP858754 |
| 4B-23-07.sqn | 4B-23-07 | PP858755 |
| 4B-23-19.sqn | 4B-23-19 | PP858756 |
| 4B-23-12.sqn | 4B-23-12 | PP858757 |
| 4B-23-13.sqn | 4B-23-13 | PP858758 |
| Z-22-103.sqn | Z-22-103 | PP858759 |
| 4B-23-15.sqn | 4B-23-15 | PP858760 |
| Z-17-046.sqn | Z-17-046 | PP858761 |
| H-23-011Sk_12.sqn | H-23-011Sk_12 | PP858762 |
| 4B-23-11.sqn | 4B-23-11 | PP858763 |
| 4B-22-41.sqn | 4B-22-41 | PP858764 |
| 4B-23-04.sqn | 4B-23-04 | PP858765 |
| Z-22-121.sqn | Z-22-121 | PP858766 |
| 4A-22-203.sqn | 4A-22-203 | PP858767 |
| 4B-23-16.sqn | 4B-23-16 | PP858768 |
| Z-14-098.sqn | Z-14-098 | PP858769 |
| 4B-22-39.sqn | 4B-22-39 | PP858770 |
| 4B-23-03.sqn | 4B-23-03 | PP858771 |
| 4B-22-37.sqn | 4B-22-37 | PP858772 |
| 4B-22-45.sqn | 4B-22-45 | PP858773 |
| 4B-23-17.sqn | 4B-23-17 | PP858774 |
| 4B-22-42.sqn | 4B-22-42 | PP858775 |
| Z-18-224.sqn | Z-18-224 | PP858776 |
