# Supplementary material for: Combining genomics and epidemiology to investigate a zoonotic outbreak of rabies in Romblon Province, Philippines
Source: Nat Commun. 2024 Dec 30;15:10753. doi: 10.1038/s41467-024-54255-5 (PMC11685615; doi:10.1038/s41467-024-54255-5)
Supplement: Supplementary file 2 — Description of Additional Supplementary Files [file 41467_2024_54255_MOESM2_ESM.pdf]

Description of Additional Supplementary Files

File Name: Supplementary Movie S1
Description: **Animation of outbreak transmission chains.** Monthly confirmed and probable dog cases coloured by genetic lineage (as per Fig. 4) shown together with the map of inferred transmission links. Squares represent sequenced cases, and circles unsequenced (unsampled) cases, except for case 45 which was not sequenced but assigned to lineage 2 based on its epidemiological link to the sequenced human case (not shown). The illustrated chains are from the consensus transmission tree with case locations simulated in proportion to human population density and pruning by the 99th percentiles of the serial interval and dispersal kernel.
