# Supplementary material for: Combining genomics and epidemiology to investigate a zoonotic outbreak of rabies in Romblon Province, Philippines
Source: Nat Commun. 2024 Dec 30;15:10753. doi: 10.1038/s41467-024-54255-5 (PMC11685615; doi:10.1038/s41467-024-54255-5)
Supplement: Supplementary file 4 — Reporting Summary [file 41467_2024_54255_MOESM4_ESM.pdf]

nature portfolio

Corresponding author(s): Yuson, Mirava

Last updated by author(s): Oct 30, 2024

Reporting Summary

Nature Portfolio wishes to improve the reproducibility of the work that we publish. This form provides structure for consistency and transparency in reporting. For further information on Nature Portfolio policies, see our Editorial Policies and the Editorial Policy Checklist.

Statistics

For all statistical analyses, confirm that the following items are present in the figure legend, table legend, main text, or Methods section.

n/a	Confirmed	
☐	☒	The exact sample size (*n*) for each experimental group/condition, given as a discrete number and unit of measurement
☐	☒	A statement on whether measurements were taken from distinct samples or whether the same sample was measured repeatedly
☒	☐	The statistical test(s) used AND whether they are one- or two-sided<br>*Only common tests should be described solely by name; describe more complex techniques in the Methods section.*
☐	☒	A description of all covariates tested
☒	☐	A description of any assumptions or corrections, such as tests of normality and adjustment for multiple comparisons
☐	☒	A full description of the statistical parameters including central tendency (e.g. means) or other basic estimates (e.g. regression coefficient) AND variation (e.g. standard deviation) or associated estimates of uncertainty (e.g. confidence intervals)
☒	☐	For null hypothesis testing, the test statistic (e.g. *F*, *t*, *r*) with confidence intervals, effect sizes, degrees of freedom and *P* value noted<br>*Give P values as exact values whenever suitable.*
☒	☐	For Bayesian analysis, information on the choice of priors and Markov chain Monte Carlo settings
☐	☒	For hierarchical and complex designs, identification of the appropriate level for tests and full reporting of outcomes
☒	☐	Estimates of effect sizes (e.g. Cohen's *d*, Pearson's *r*), indicating how they were calculated

Our web collection on statistics for biologists contains articles on many of the points above.

Software and code

Policy information about availability of computer code

Data collection	ODK Collect was used to encode human and animal rabies case information.
Data analysis	Epidemiological and genomic surveillance data was analysed using R version 4.3.0, and transmission tree analyses were analysed using R version 4.1.3.. Further phylogenetic analysis was completed using MAFFT (v7.520) for aligning whole genome sequences, and FastTree v2.1.11 with a GTR+Gamma20 model for constructing maximum likelihood trees. The BactDating R Package's initRoot function was used to root the maximum likelihood tree according to the best root-to-tip correlation, followed by gotree (v0.4.5) for pruning and R-wrapper for lsd2 (Rlsd2) for estimating the evolutionary rate. PastML (v1.9.42) was used to perform ancestral character reconstructions on the dated tree. Phylogenetic tree annotation and visualisation was performed in R with the ggtree package. Transmission trees were constructed using the treerabid R package v1.0.1, converted to graph objects using igraph (v1.3.5) and visualised using ggraph (v2.1.0). In this analysis, we used the dplyr, tidyr, magrittr, readr, lubridate, data.table, foreach, iterators, doParallel and doRNG packages for data processing. Additional R packages used to enhance visualisations were ggplot2 (v3.5.1), ggthemes (v5.1.0), cowplot (v1.1.3) and patchwork (v1.3.0). An animation of the consensus transmission tree was generated using the ggforce (v0.4.1) and animation (v2.7) R packages and the FFmpeg software (v6.1.1). R packages sf and raster were used for processing geographical data. Population density estimates were obtained from WorldPop's 2018 unconstrained model. The adegenet, adephylo, ape and dendextend packages were used to identify genetic clusters and visualise them on a heatmap. Code and data are all provided in the Github repository: https://github.com/boydorr/outbreak_romblon_PHL/.

For manuscripts utilizing custom algorithms or software that are central to the research but not yet described in published literature, software must be made available to editors and reviewers. We strongly encourage code deposition in a community repository (e.g. GitHub). See the Nature Portfolio guidelines for submitting code & software for further information.

Data

Policy information about availability of data

All manuscripts must include a data availability statement. This statement should provide the following information, where applicable:

- Accession codes, unique identifiers, or web links for publicly available datasets
- A description of any restrictions on data availability
- For clinical datasets or third party data, please ensure that the statement adheres to our policy

The rabies surveillance and phylogenetic data used in this study are available in the Github repository:
https://github.com/boydorr/outbreak_romblon_PHL
The genome sequence data are provided in Supplementary Table S1, while the GenBank accession numbers are provided in Supplementary Table S2.

Research involving human participants, their data, or biological material

Policy information about studies with human participants or human data. See also policy information about sex, gender (identity/presentation), and sexual orientation and race, ethnicity and racism.

Reporting on sex and gender	Sex and gender of human rabies biospecimens were included in case details but are known only by the authors and were not reported in the study for confidentiality purposes.
Reporting on race, ethnicity, or other socially relevant groupings	Race and ethnicity of human rabies biospecimens were included in case details but are known only by the authors and were not reported in the study for confidentiality purposes.
Population characteristics	The two human rabies cases were referred to only by their age group as well as their location at the time of transmission (i.e. Tablas Island, Romblon Province, Philippines):<br><br>Child - <12 year-old (Genbank accession number H-23-011Sk_12 / PP858762) from Santa Maria municipality<br>Elderly - >70 year-old elderly from Odiongan municipality<br><br>They were both initially diagnosed based on symptoms and history of exposure, followed by laboratory confirmation through nested polymerase chain reaction (PCR) of collected post-mortem sample (saliva and nuchal neck skin scrape).
Recruitment	All patients in Romblon that were classified as human rabies cases after laboratory confirmation were included in the study.
Ethics oversight	Ethical review was secured from Research Institute for Tropical Medicine (RITM) ethical review board (2019-023) and University of Glasgow, Medical, Veterinary & Life Sciences ethics committee (200190123).

Note that full information on the approval of the study protocol must also be provided in the manuscript.

Field-specific reporting

Please select the one below that is the best fit for your research. If you are not sure, read the appropriate sections before making your selection.

☒ Life sciences ☐ Behavioural & social sciences ☐ Ecological, evolutionary & environmental sciences

For a reference copy of the document with all sections, see nature.com/documents/nr-reporting-summary-flat.pdf

Life sciences study design

All studies must disclose on these points even when the disclosure is negative.

Sample size	This is not applicable since we are describing an outbreak which took place in the Phillipines and sample size was dictated by the number of cases detected and sequencing capacity.
Data exclusions	No data were excluded from the analyses.
Replication	As experimental findings do not exist for this study, replication was not considered necessary. All code and data related to the findings of this study are provided in the Github repository where they can be validated and reproduced: https://github.com/RabiesResearch/outbreak_romblon_PH
Randomization	Randomization was not relevant to this study as no treatment or control groups were used.
Blinding	Blinding was not relevant to this study as no treatment or control groups were used.

Reporting for specific materials, systems and methods

We require information from authors about some types of materials, experimental systems and methods used in many studies. Here, indicate whether each material, system or method listed is relevant to your study. If you are not sure if a list item applies to your research, read the appropriate section before selecting a response.

Materials & experimental systems

n/a	Involved in the study
☒	☐ Antibodies
☒	☐ Eukaryotic cell lines
☒	☐ Palaeontology and archaeology
☐	☒ Animals and other organisms
☒	☐ Clinical data
☒	☐ Dual use research of concern
☒	☐ Plants

Methods

n/a	Involved in the study
☒	☐ ChIP-seq
☒	☐ Flow cytometry
☒	☐ MRI-based neuroimaging

Animals and other research organisms

Policy information about studies involving animals; ARRIVE guidelines recommended for reporting animal research, and Sex and Gender in Research

Laboratory animals	The study did not involve laboratory animals.
Wild animals	Sample collection was performed on dead animals (dogs, cats and other mammals regardless of sex or age) that were considered suspicious for rabies after an animal investigation was conducted by a trained animal health worker. While some animals were alive while diagnosed as probable rabies cases, they were not euthanised, but instead quarantined at their owner's residence and monitored until they died. Only then were samples collected. Age was not considered in this study design as it did not influence test results. Samples from juvenile and adult animals were retrieved so long as they were suspected to be rabid.
Reporting on sex	Sex was not considered in this study design as it did not influence test results. Samples from male and female animals were retrieved so long as they were suspected to be rabid.
Field-collected samples	Samples collected from the field were properly stored in iceboxes and maintained at appropriate temperatures while in transport to the laboratory for confirmatory testing. If samples could not be transported to laboratories immediately, they were stored temporarily in a freezer.
Ethics oversight	Ethical review was secured from Research Institute for Tropical Medicine (RITM) ethical review board (2019-023) and University of Glasgow, Medical, Veterinary & Life Sciences ethics committee (200190123).

Note that full information on the approval of the study protocol must also be provided in the manuscript.

Plants

Seed stocks	Not applicable
Novel plant genotypes	Not applicable
Authentication	Not applicable
